# Intelligent and Efficient Quarantine

**Published:** 1878-02

**Authors:** 


### Intelligent and Efficient Quarantine.

The report of Dr. Vanderpoel, Health Officer of the Port of New York, to the Commissioners of Quarantine, contains some interesting and suggestive facts. It would appear, as far as these facts will allow us to draw any conclusions, that the problem has been solved of securing effective quarantine at the same time that the interests of commercial circles have been amply considered and satisfactorily protected. Heretofore the existence of yellow fever in the port of New York would have been considered a public calamity, and would have called forth the most strenuous exertions on the part of our health authorities to make the quarantine complete and absolute.

Now, however, we are informed by the Health Officer that the arrival of vessels, having had cases of yellow fever on board, is an event much of the time of weekly occurrence. "During at least five months of the year little or no significance is given to the occurrence, and vessels are given an unrestrained pratique, as if no sickness had occurred." And again, "During the period when the transmission of yellow fever is to be feared, and when the examination of suspected vessels is made, 1,143 vessels were at the station in Lower Bay. Of this number 183 arrived from ports known to be infected; 44 vessels arrived from such ports, having sickness during their stay there, on passage or on arrival here; 140 cases of yellow fever occurred upon them when in an infected port, of whom 36 died; 14 on passage, of whom 12 died; 25 in quarantine, of whom 13 died. Of 7 of the 13 deaths 2 died before reaching hospital and 5 were moribund on arrival at hospital; there were 28 cases in hospital, 25 of which were yellow fever; excluding the 7 above mentioned would leave 18 cases, among whom were 5 deaths, it being about the ordinary percentage for the hospital. The remaining death was from Chagres fever." These are significant facts taken in connection with the statement that no vessels were detained in quarantine longer than for purposes of disinfection, and conclusively prove that sanitary measures and commerce need not be antagonistic, but that the former in this instance was a positive aid to the latter.

This system of quarantine, which was first organized by Dr. Vanderpoel, is for all practical purposes as near perfection as it can be, and after a successful trial of six years in the largest port of entry in the United States it presents itself with all the force of an established principle.—*Medical Record.*

